# Supplementary material for: Clinical Efficacy of Hypofractionated Proton Beam Therapy for Intrahepatic Cholangiocarcinoma
Source: Cancers (Basel). 2022 Nov 12;14(22):5561. doi: 10.3390/cancers14225561 (PMC9688899; doi:10.3390/cancers14225561)
Supplement: Supplementary file 1 [file cancers-14-05561-s001.zip › cancers-2022287-supplementary.pdf]

Supplementary Materials

Supplementary Figures

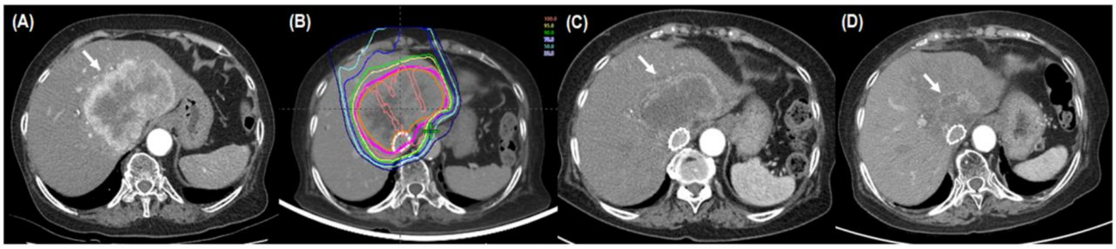

Figure S1. An example of proton beam therapy (PBT) for intrahepatic cholangiocarcinoma (IHCC). (A) Computed tomography (CT) scans at diagnosis showing the tumor (arrow). (B) Plan CT scans for proton beam therapy (PBT) showing dose line. (C and D) CT scans at 6 and 12 months, respectively, after PBT showing marked shrinkage of the tumor (arrow).

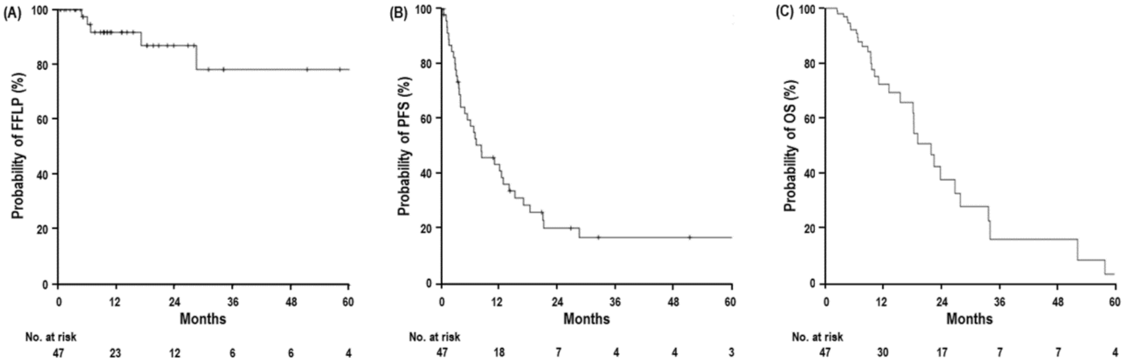

Figure S2. Freedom from local progression (FFLP) (A), progression-free survival (PFS) (B), and overall survival (OS) (C) curves in intrahepatic cholangiocarcinoma patients treated with proton beam therapy.

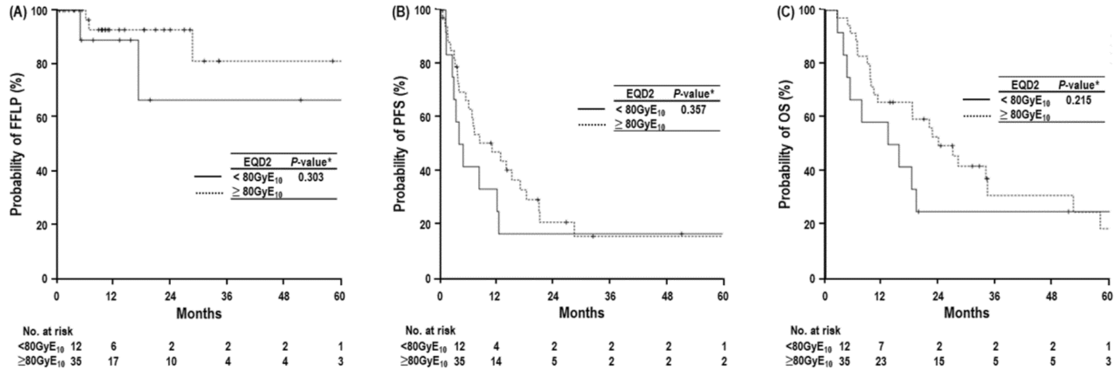

Figure S3. Freedom from local progression (FFLP) (A), progression-free survival (PFS) (B), and overall survival (OS) (C) according to total dose (EQD2) of proton beam therapy.

Abbreviations: EQD2, equivalent dose in 2 Gy fractions (EQD2=total dose×[(fraction dose+ α/β)/(2+α/β)], α/β = 10); and GyE, gray equivalent (GyE=proton physical dose [in gray]×relative biologic effectiveness [1.1]).

*log-rank test.

Supplementary Tables

Table S1. Treatment modalities prior or after proton beam therapy (PBT)

Details of treatment modalities		Distribution, n (%)
Pre-Tx to PBT site(s)	No	26 (55.3)
	Yes	21 (44.7)
	SAT*	20 (42.6)
	TACE	1 (2.1)
Post-Tx after PBT	No	23 (48.9)
	Yes	24 (51.1)
	SAT†	20 (42.6)
	SAT† + SR	3 (6.4)
	LT	1 (2.1)

Abbreviations: n, number of patients; Tx, treatment; TACE, transarterial chemoembolization; SAT, systemic anticancer therapy; SR, surgical resection; and LT, liver trasplantation.

*Gemcitabine and cisplatin (n=13), gemcitabine, cisplatin and pembrolizumab (n=2), gemcitabine (n=2), capecitabine and cisplatin (n=1), oxaliplatin, irinotecan and tegafur/gimeracil/oteracil (n=1), and gemcitabine, cisplatin and albumin-bound paclitaxel (n=1).

†Gemcitabine and cisplatin (n=15), gemcitabine (n=3), gemcitabine, cisplatin and albumin-bound paclitaxel (n=2), capecitabine (n=1), 5-fluorouracil, irinotecan, leucovorin and oxaliplatin (n=1), and nivolumab (n=1).

Table S2. Summary of studies intrahepatic cholangiocarcinoma treated with radiotherapy

Author	Year	Study design	Type of RT	n	Total dose (Gray/fractions)	FFLP	OS	AE rate*
Zeng et al.[1]	2006	Retro	RT	38	30 - 60 / 15 - 30	-	50.1% (1yr)<br>11.8% (2yr)	5.3%
			No RT	37	-	-	24.8% (1yr)<br>5.5% (2yr)	-
Chen et al.[2]	2010	Retro	RT	35	30 - 60 / 15 - 30	-	9.5m (med)<br>38.5% (1yr)<br>16.4% (2yr)	2.9%
			No RT	49	-	-	5.1 (med)<br>9.6% (1yr)<br>4.9% (2yr)	-
Kim et al.[3]	2013	Retro	CRT	25	25 - 60 / 12 - 30	-	9.3m (med)<br>30.4% (1yr)	24.0%
			SAT	67		-	6.2m (med)<br>22.4% (1yr)	10.4%
Tse et al.[4]	2008	Phase I	SBRT	10	28.2 - 48 / 6	65% (1yr)	15m (med)	12%
Barney et al.[5]	2012	Retro	SBRT	10	45 - 60 / 3 - 5	100% (1yr)	15.5m (med)<br>73% (1yr)	20%
Jung et al.[6]	2014	Retro	SBRT	58	15 - 60 / 1 - 5	85% (1yr)<br>75% (2yr)	10m (med)<br>45% (1yr)<br>20% (2yr)	10%
Mahadevan et al.[7]	2015	Retro	SBRT	34	24 - 45 / 3 - 5	88% (1yr)	17m (med)<br>58% (1yr)<br>31% (2yr)	12%
Sandler et al.[8]	2016	Retro	SBRT	31	25 - 50 / 5	78% (1yr)<br>47% (2yr)	15.7m (med)<br>59% (1yr)<br>33% (2yr)	16%
Gkika et al.[9]	2017	Retro	SBRT	37	21 - 66 / 3 - 12	78% (1yr)<br>58% (2yr)	14m (med)<br>57% (1yr)<br>25% (2yr)	9%
Tao et al.	2016	Retro	IMRT/PBT	79	30 – 100 / 3 - 30	81% (1yr)	30m (med)<br>87% (1yr)<br>61% (2yr)	6.3%
Smart et al.[10]	2020	Retro	HF-RT/PBT	66	37.5 – 67.5 / 15	84% (2yr)	58% (2yr)	11%
Makita et al.[11]	2014	Retro	PBT	18	50.6 – 80 / 15 - 40	67.7% (1yr)	49.0% (1yr)	22.2%
Onkawa et al.[12]	2015	Retro	PBT	20	55 – 79.2 / 10 - 16	88% (1yr)<br>60% (2yr)	27.5m† / 9.6m‡ (med)	0%
Hong et al. [13]	2016	Phase II	PBT	37	15.1 – 67.5 / 15	94.1% (2yr)	22.5m (med)<br>46.5% (2yr)	7.7%
Hung et al.[14]	2019	Retro	PBT	30	39.6 – 73.4 / 10 - 24	88% (1yr)	19.3m (m)<br>83% (1yr)<br>32% (2yr)	17.4%
Shimizu et al.[15]	2019	Retro	PBT	42	46.6 – 74 / 12 - 37	97.3% (1yr)<br>68.4% (2yr)	15.0m (med)<br>60.3% (1yr)<br>41.4% (2yr)	0%
Present study	2022	Retro	PBT	47	45 – 80 / 10	91.7% (1yr)<br>86.9% (2yr)	21.9m (med)<br>63.8% (1yr)	8.5%

	42.7% (2yr)

Abbrevations:n, number of patients; RT, radiotherapy; FFLP, freedom from local progression; OS, overall survival; AE, adverse events; Retro, retrospective; CRT, chemoradiotherapy; SBRT, stereotactic body RT; IMRT, intensity-modulated RT; HF-RT, hypofractionated RT; PBT, proton beam therapy; med, median; yr, year; the others are same as Table S1.

[*]rate of ≥ grade 3 adverse events or major adverse events.

[†]Curative group

[‡]Palliative group

References

1. Zeng, Z.C.; Tang, Z.Y.; Fan, J.; Zhou, J.; Qin, L.X.; Ye, S.L.; Sun, H.C.; Wang, B.L.; Li, D.; Wang, J.H.; et al. Consideration of the role of radiotherapy for unresectable intrahepatic cholangiocarcinoma: a retrospective analysis of 75 patients. *Cancer J* **2006**, *12*, 113-122.
2. Chen, Y.X.; Zeng, Z.C.; Tang, Z.Y.; Fan, J.; Zhou, J.; Jiang, W.; Zeng, M.S.; Tan, Y.S. Determining the role of external beam radiotherapy in unresectable intrahepatic cholangiocarcinoma: a retrospective analysis of 84 patients. *BMC Cancer* **2010**, *10*, 492, doi:10.1186/1471-2407-10-492.
3. Kim, Y.I.; Park, J.W.; Kim, B.H.; Woo, S.M.; Kim, T.H.; Koh, Y.H.; Lee, W.J.; Kim, C.M. Outcomes of concurrent chemoradiotherapy versus chemotherapy alone for advanced-stage unresectable intrahepatic cholangiocarcinoma. *Radiat Oncol* **2013**, *8*, 292, doi:10.1186/1748-717X-8-292.
4. Tse, R.V.; Hawkins, M.; Lockwood, G.; Kim, J.J.; Cummings, B.; Knox, J.; Sherman, M.; Dawson, L.A. Phase I study of individualized stereotactic body radiotherapy for hepatocellular carcinoma and intrahepatic cholangiocarcinoma. *J Clin Oncol* **2008**, *26*, 657-664, doi:10.1200/JCO.2007.14.3529.
5. Barney, B.M.; Olivier, K.R.; Miller, R.C.; Haddock, M.G. Clinical outcomes and toxicity using stereotactic body radiotherapy (SBRT) for advanced cholangiocarcinoma. *Radiat Oncol* **2012**, *7*, 67, doi:10.1186/1748-717X-7-67.
6. Jung, D.H.; Kim, M.S.; Cho, C.K.; Yoo, H.J.; Jang, W.I.; Seo, Y.S.; Paik, E.K.; Kim, K.B.; Han, C.J.; Kim, S.B. Outcomes of stereotactic body radiotherapy for unresectable primary or recurrent cholangiocarcinoma. *Radiat Oncol J* **2014**, *32*, 163-169, doi:10.3857/roj.2014.32.3.163.
7. Mahadevan, A.; Dagoglu, N.; Mancias, J.; Raven, K.; Khwaja, K.; Tseng, J.F.; Ng, K.; Enzinger, P.; Miksad, R.; Bullock, A.; et al. Stereotactic Body Radiotherapy (SBRT) for Intrahepatic and Hilar Cholangiocarcinoma. *J Cancer* **2015**, *6*, 1099-1104, doi:10.7150/jca.13032.
8. Sandler, K.A.; Veruttipong, D.; Agopian, V.G.; Finn, R.S.; Hong, J.C.; Kaldas, F.M.; Sadeghi, S.; Busuttil, R.W.; Lee, P. Stereotactic body radiotherapy (SBRT) for locally advanced extrahepatic and intrahepatic cholangiocarcinoma. *Adv Radiat Oncol* **2016**, *1*, 237-243, doi:10.1016/j.adro.2016.10.008.
9. Gkika, E.; Hallauer, L.; Kirste, S.; Adebahr, S.; Bartl, N.; Neeff, H.P.; Fritsch, R.; Brass, V.; Nestle, U.; Grosu, A.L.; et al. Stereotactic body radiotherapy (SBRT) for locally advanced intrahepatic and extrahepatic cholangiocarcinoma. *BMC Cancer* **2017**, *17*, 781, doi:10.1186/s12885-017-3788-1.
10. Smart, A.C.; Goyal, L.; Horick, N.; Petkovska, N.; Zhu, A.X.; Ferrone, C.R.; Tanabe, K.K.; Allen, J.N.; Drapek, L.C.; Qadan, M.; et al. Hypofractionated Radiation Therapy for Unresectable/Locally Recurrent Intrahepatic Cholangiocarcinoma. *Ann Surg Oncol* **2020**, *27*, 1122-1129, doi:10.1245/s10434-019-08142-9.
11. Makita, C.; Nakamura, T.; Takada, A.; Takayama, K.; Suzuki, M.; Ishikawa, Y.; Azami,

Y.; Kato, T.; Tsukiyama, I.; Kikuchi, Y.; et al. Clinical outcomes and toxicity of proton beam therapy for advanced cholangiocarcinoma. *Radiat Oncol* **2014**, *9*, 26, doi:10.1186/1748-717X-9-26.

12. Ohkawa, A.; Mizumoto, M.; Ishikawa, H.; Abei, M.; Fukuda, K.; Hashimoto, T.; Sakae, T.; Tsuboi, K.; Okumura, T.; Sakurai, H. Proton beam therapy for unresectable intrahepatic cholangiocarcinoma. *J Gastroenterol Hepatol* **2015**, *30*, 957-963, doi:10.1111/jgh.12843.
13. Hong, T.S.; Wo, J.Y.; Yeap, B.Y.; Ben-Josef, E.; McDonnell, E.I.; Blaszkowsky, L.S.; Kwak, E.L.; Allen, J.N.; Clark, J.W.; Goyal, L.; et al. Multi-Institutional Phase II Study of High-Dose Hypofractionated Proton Beam Therapy in Patients With Localized, Unresectable Hepatocellular Carcinoma and Intrahepatic Cholangiocarcinoma. *J Clin Oncol* **2016**, *34*, 460-468, doi:10.1200/JCO.2015.64.2710.
14. Hung, S.P.; Huang, B.S.; Hsieh, C.E.; Lee, C.H.; Tsang, N.M.; Chang, J.T.; Chen, J.S.; Chou, W.C.; Tseng, J.H.; Hong, J.H. Clinical Outcomes of Patients With Unresectable Cholangiocarcinoma Treated With Proton Beam Therapy. *Am J Clin Oncol* **2020**, *43*, 180-186, doi:10.1097/COC.0000000000000646.
15. Shimizu, S.; Okumura, T.; Oshiro, Y.; Fukumitsu, N.; Fukuda, K.; Ishige, K.; Hasegawa, N.; Numajiri, H.; Murofushi, K.; Ohnishi, K.; et al. Clinical outcomes of previously untreated patients with unresectable intrahepatic cholangiocarcinoma following proton beam therapy. *Radiat Oncol* **2019**, *14*, 241, doi:10.1186/s13014-019-1451-5.
